# Rewarding distractor context versus rewarding target location: A commentary on Tseng and Lleras (2013)

**DOI:** 10.3758/s13414-014-0668-5

**Published:** 2014-03-25

**Authors:** Bernhard Schlagbauer, Thomas Geyer, Hermann J. Müller, Michael Zehetleitner

**Affiliations:** 1 Department Psychologie, Ludwig-Maximilians-Universität München, Munich, Germany; 2 School of Psychology, Birkbeck College, University of London, London, UK; 3 Department Psychologie, Lehrstuhl für Allgemeine und Experimentelle Psychologie, Ludwig-Maximilians-Universität München, Leopoldstraße 13, 80802 München, Germany

**Keywords:** Attention, Visual search, Contextual cueing, Reward, Probability cueing

## Abstract

The influence of reward on cognitive processes including visual perception, spatial attention, and perceptual learning has become an increasingly important field of study in recent years. For example, Tseng and Lleras (*Attention, Perception, & Psychophysics*, *75*(2), 287–298, 2013) investigated whether reward has an effect on implicit learning of target–distractor arrangements in visual search—that is, contextual cueing (Chun & Jiang *Cognitive Psychology*, *36*(1), 28–71, 1998). They found that reward expedited the development of the cueing effect—that is, the reaction time difference between repeated and nonrepeated displays. However, their analysis did not account for potential effects of reward on the learning of individual target locations—that is, probability cueing (Jiang, Swallow, & Rosenbaum *Journal of Experimental Psychology. Human Perception and Performance*, *39*, 285–297, 2013). The present study was a replication of Tseng and Lleras (*Attention, Perception, & Psychophysics*, *75*(2), 287–298, 2013) that took into account reward effects on configural and locational learning, as well. We found that reward led to performance gains even in baseline (“new”) displays, which contained only repeated target, but not distractor, locations. Furthermore, contextual cueing was smaller, and not larger, in high- than in low-reward trials. We concluded that reward modulates probability, and not contextual, cueing, and that this mechanism can account for the findings of Tseng and Lleras.

Humans are severely limited in their ability to process the entire perceptual array (Simons & Rensink, 2005). The mechanisms that are important for overcoming this limitation are reflexively—that is, bottom-up—and intentionally—that is, top-down—guided visual attention (Corbetta & Shulman, 2002), in addition to guidance based on motivational heuristics (Della Libera & Chelazzi, 2006). Thus, the central question is which particular aspects of a scene are worth looking at, and one of the most prominent motivational factors determining worthiness is reward. A number of recent studies have shown that currently rewarded items guide attention (Hickey, Chelazzi, & Theeuwes, 2010a, 2010b, 2011), or that items that were rewarded in the past can continue to have attention-attracting power, even if they are no longer rewarded on current trials (Anderson, Laurent, & Yantis, 2011). These findings suggest that reward improves the deployment of visual selective attention by associating stimulus features with high reward value.

Besides consistent reward, other regularities in the visual environment can also be learned by an observer to facilitate search behavior. Especially, spatial invariances in a visual scene are known to serve as cues to action-relevant objects. Two types of regularities are of particular interest in the present study: the spatial layout of nontarget, or context, elements—that is, contextual cueing (Chun & Jiang, 1998)—and the probability for positional repetitions of target elements—that is, probability cueing (Geng & Behrmann, 2005; Jiang, Swallow, & Rosenbaum, 2013). *Probability cueing* refers to the capability of the visual system to perform statistical inferences about the likelihood of a given target position. If the target in visual search (Wolfe, 1998) is more likely to appear at a given location, this position is learned and prioritized over other locations. As a result, reaction times (RTs) are faster on high than on low target position probability trials (Jiang et al., 2013).

*Contextual cueing* refers to the observation of expedited RTs to repeated, relative to non-repeated—that is, novel, target–distractor arrangements in visual search. Contextual cueing emerges after approximately five repetitions of a given repeated display (Chaumon, Schwartz, & Tallon-Baudry, 2009; Chun & Jiang, 1998) and remains observable even after several days (Chun & Jiang, 2003). Although contextual cueing can lead to strong RT gains, participants are nevertheless unable to distinguish repeated from nonrepeated displays (Chun & Jiang, 1998). This discrepancy between indirect (RT) and direct (explicit recognition) measures has led to the proposal of implicit contextual cueing (but see, e.g., Schlagbauer, Müller, Zehetleitner, & Geyer, 2012, or Smyth & Shanks, 2008, for discrepant views). Moreover, there is evidence that the cueing effect can exert its influences at various visual-processing stages, including preattentive and postselective stages, as well (pro preattentive: Geyer, Zehetleitner, & Müller, 2010; Johnson, Woodman, Braun, & Luck, 2007; pro postselective: Kunar, Flusberg, Horowitz, & Wolfe, 2007).

Although contextual cueing is an implicit—that is, cognitively impenetrable—effect, Tseng and Lleras (2013) recently asked whether motivational factors like the delivery of monetary reward can nevertheless influence the cueing effect. They hypothesized that rewarding the distractors’ arrangement in a contextual-cueing task may enhance configuration-specific learning. The authors used the “standard” contextual-cueing paradigm, introduced by Chun and Jiang (1998), which requires observers to search for a T-shaped target letter among L-shaped distractor letters and subsequently to report the target’s orientation (left- vs. right-tilted). Importantly, half of the trials contained repeated target–distractor arrangements. Additionally, the authors introduced different levels of reward—namely, gaining points (reward condition), losing points (penalty condition), or no reward (no-outcome condition). Of relevance to the present study is the authors’ finding of reward-based contextual cueing: For rewarded relative to nonrewarded target–distractor arrangements, the cueing effect became manifest in earlier experimental epochs (but reward did not increase the overall size of the cueing effect).

Tseng and Lleras’s (2013) design, however, had one shortcoming. As we elaborated earlier, there are two different types of spatial learning effects in repeated visual search: probability cueing and contextual cueing. One important methodological aspect of contextual-cueing studies is to control for target location repetition effects, and therefore to isolate effects of repeated distractor from repeated target positions (cf. Chun & Jiang, 1998). Since the repetition of a whole search display in the repeated-display condition includes both identically positioned target and distractor items, observers could use either of these cues to facilitate their search. Consequently, in standard contextual-cueing experiments, target positions are kept constant across nonrepeated displays as well, so that the only crucial difference between search conditions is the spatial layout.

Tseng and Lleras (2013) compared repeated (old) displays associated with three different reward outcomes to nonrepeated (new) displays, but with the latter averaged across the three reward conditions. Specifically, a given old display was either positively rewarded (“rewarding context”: six out of 12 old displays), negatively rewarded (“penalizing context”: three old displays), or not associated with any reward (“no outcome”: three old displays)—importantly, with the reward association being kept constant across the entire experiment. A similar reward scheme was applied in the “new,” baseline condition (which consisted of 12 repeated target, amongst nonrepeated distractor, locations); but, for their data analysis, Tseng and Lleras merged all RTs on positive-, negative-, and neutral-reward trials into a single new (“mean”) condition, because “reward occurred after the response and new displays were never repeated” (p. 290). However, collapsing RTs into a single new condition is potentially problematic, since this would (1) hide any effects of the reward manipulation on target probability learning in the new (“mean”) condition, and thus, (2) confound the effects of reward on configural versus target probability learning in the “old” condition. Arguably, in order to isolate the effect of reward on the learning of distractor contexts (unconfounded by target probability learning), a full factorial design would be necessary, in which reward was also manipulated in the “new” condition and RT performance was compared for corresponding levels of reward between the old and new conditions. Thus, given that Tseng and Lleras did not examine their data in this manner, it is possible that their reward manipulation had an effect on target location learning—in addition to, or instead of, configural learning.

To examine for an influence of reward-based target location learning in Tseng and Lleras’s (2013) paradigm, we implemented a full factorial contextual-cueing experiment manipulating context (old, new) and reward (high, low). In each half of the trials, target locations were associated with high and, respectively, with low reward. Orthogonal to this, distractor configurations were either repeated (old displays) or newly generated (new displays), independently of the level of reward associated with particular target locations. Thus, we created two levels of reward (targets appearing at locations associated with high vs. low reward) for both old and new displays. This allowed us to assess (1) whether reward influences target position learning (by comparing RTs between high- and low-reward trials in both the old and new conditions) and (2) whether reward influences configural learning (by comparing RTs between old and new displays in both high- and low-reward trials).

In summary, an effect of reward in the new condition would be attributable only to reward-based improvements of location-specific learning. However, if reward enhances configuration-specific learning in repeated visual search, as was proposed by Tseng and Lleras (2013), one would expect contextual cueing to be more marked—that is, to arise earlier—for high- than for low-reward trials. Of course, the latter prediction does not exclude the possibility of reward effects on both configuration- and location-specific learning in repeated visual search.

## Method

### Participants

As in Experiment 1 of Tseng and Lleras (2013), a total of 25 observers took part in the experiment (16 female, nine male; mean age: 25.4 years). All participants reported normal or corrected-to-normal vision and were naïve as to the purpose of the study. They provided written informed consent prior to the experiment and received payment depending on their performance, with a minimum payment of €8 (~USD10.7) for a 1-h experimental session.

### Apparatus and stimuli

The experiment was controlled by a Dell PC running MATLAB with the Psychophysics Toolbox extension for stimulus presentation (Brainard, 1997; Pelli, 1997). Participants were seated in front of a 24-in. CRT monitor (Mitsubishi Diamond Pro; refreshed at a rate of 120 Hz) at a viewing distance of approximately 80 cm. The search displays consisted of 12 dark gray items (1.0 cd/m^2^; one target and 11 distractors) presented against a light gray background (25.4 cd/m^2^). All stimuli extended 0.35º of visual angle in width and height. The items were arranged along four (invisible) concentric circles around the display center (radii: 1.74º, 3.48º, 5.22º, and 6.96º), with the target always being positioned on the third circle from the display center. The “T” target was oriented randomly either 90º or 270º from the vertical midline (“L” distractors: 0º, 90º, 180º, or 270º).

### Trial procedure

Each trial started with the presentation of a fixation cross (0.35 × 0.35º, 1.0 cd/m^2^) at the center of the screen for 500 ms, followed by a blank interval of 200 ms; thereafter, the search displays appeared. The participants’ task was to respond as quickly and accurately as possible to the orientation of the target. When the target was oriented to the left (vs. the right), they pressed the left (vs. the right) arrow key of the computer keyboard. Displays were visible until a response was made, or maximally for 2,000 ms. Following observers’ search task response and another blank interval of 200 ms, error feedback and feedback indicating monetary reward was given. This information stayed on the screen until participants pressed the down arrow key (but for at least 750 ms), which triggered the start of the next trial (the blank intertrial interval was 500 ms). Participants performed 20 blocks of 24 trials each, yielding a total of 480 trials.

### Design

The “old” condition contained 12 randomly arranged target–distractor configurations, generated at the beginning of the experiment. These were repeatedly presented on randomly selected trials throughout the search task, with the restriction that each repeated display was shown only once per block. Displays in the “new” condition were generated online on a given trial. On half of the trials an old arrangement was presented, and a new arrangement on the other half. To equate target location repetition effects between the two types of displays, the target appeared equally often at each of 24 possible locations throughout the experiment: 12 locations were used for repeated, and the other 12 for nonrepeated, displays. Note that for both old and new displays, the target was equally likely to appear in any of the four display quadrants. Furthermore, item density was kept constant across the four display quadrants (each quadrant contained three items).

Half of the displays were rewarded with 5 cents (high-reward trials), the other half with 1 cent (low-reward trials). After their search task response, participants received feedback about their current reward (5 cents, 1 cent, or 0 cents, in the case of an error or time-out) and how much money they had gathered thus far. Participants were not informed—either in the beginning or on a trial-by-trial basis—about the details of the experimental reward manipulation. Importantly, the reward was assigned consistently: The very same 12 target positions were associated with either high or low reward, six of which were embedded in repeated distractor arrangements (old displays), and the other six in random distractor arrangements (new displays).

## Results

The data analysis was performed using R (R Development Core Team, 2013). In order to obtain reasonable estimates of the contextual-cueing effect, the data of five consecutive blocks were pooled into one “epoch” (see Chun & Jiang, 1998), resulting in four experimental epochs. Incorrect trials (including erroneous responses and time-outs) were discarded from the analysis (mean error rate: 5.98%). A 2 (context) × 2 (reward) × 4 (epoch) repeated measures analysis of variance (ANOVA) revealed that (only) the effect of epoch was significant [*F*(3, 72) = 9.3, *p* < .001, *η*_p_^2^ = .280; i.e., fewer response errors occurred in later epochs]. Furthermore, “extreme” RTs outside ±2.5 standard deviations of the individual mean RTs were also excluded from the analysis (overall, 1.90% of trials).

### Replication of Tseng and Lleras (2013)

First, we pooled RTs across both reward conditions in the new displays and compared them with those from the old displays, separately for the high- and low-reward conditions (Tseng & Lleras, 2013). A two-way repeated measures ANOVA with the factors Display Type (old displays–high reward, old displays–low reward, new displays) and Epoch (1–4) revealed main effects of both display type [*F*(2, 48) = 6.1, *p* = .004, *η*_p_^2^ = .203] and epoch [*F*(3, 72) = 41.7, *p* < .001, *η*_p_^2^ = .634]. Although the interaction was nonsignificant [*F*(6, 144) = 1.5, *p* = .183], direct tests were conducted for each individual epoch (*p* values were Holm-corrected for multiple *t* tests) in order to track the time course of contextual cueing. As expected, high-reward displays yielded a contextual-cueing effect already in the first epoch (44, 72, 90, and 104 ms in Epochs 1–4, all *p*s < .007). For low-reward displays, we observed at least some tendency for the cueing effect to develop later in time, although the effect failed to reach significance in any of the four epochs [Epoch 1: 8 ms, *t*(24) = 0.3, *p* = .366; Epoch 2: 29 ms, *t*(24) = 1.6, *p* = .189; Epoch 3: 41 ms, *t*(24) = 2.0, *p* = .120, Epoch 4: 27 ms, *t*(24) = 1.1, *p* = .263].

### Full design with rewarded new displays

**Fig. 1:**
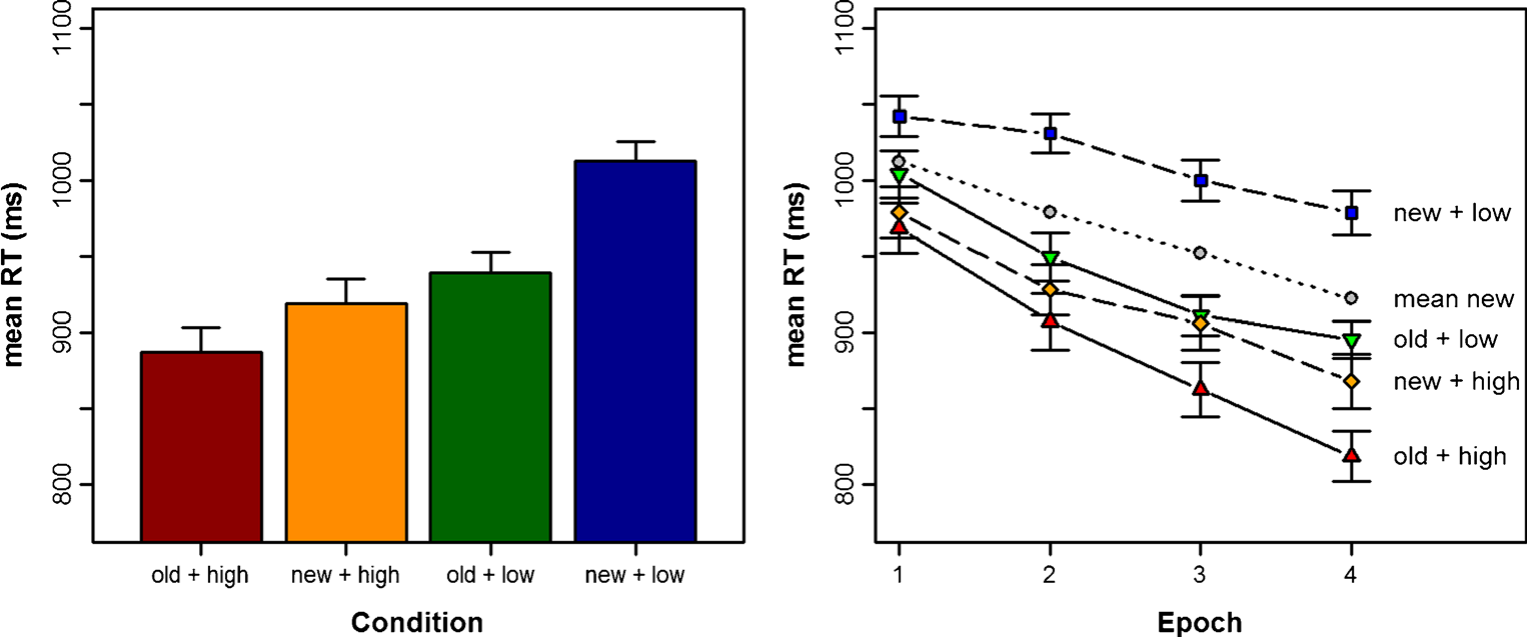
Reaction time (RT) performance. (Left) Mean RTs as a function of reward (high, low) and context (old, new). The data are collapsed across all epochs. (Right) Mean RTs as a function of reward (high, low) and context (old, new), separately for Epochs 1–4. Gray circles represent the RTs to new displays averaged across the (high, low) reward conditions (“mean new”), following Tseng and Lleras (2013)

Second, to analyze the reward effects associated with repeated target positions (in addition to those resulting from repeated distractor contexts), RTs were analyzed by means of a three-way repeated measures ANOVA with the factors Context (old vs. new), Reward (high vs. low), and Epoch (1–4). All main effects were significant: context [*F*(1, 24) = 72.3, *p* < .001, *η*_p_^2^ = .751], reward [*F*(1, 24) = 5.5, *p* = .028, *η*_p_^2^ = .185], and epoch [*F*(3, 72) = 40.1, *p* < .001, *η*_p_^2^ = .626]. Furthermore, the interactions between context and epoch [*F*(3, 72) = 3.1, *p* = .032, *η*_p_^2^ = .114] and, of most interest here, between context and reward [*F*(1, 24) = 9.3, *p* = .005, *η*_p_^2^ = .280] were significant. Further analyses were carried out to explore the latter interaction. First, a comparison of mean RTs revealed that observers responded most quickly to old displays with high reward (887 ms), with intermediate speed to new displays with high reward (919 ms), and most slowly to new displays with low reward (1,013 ms) (all *p*s < .01, Holm-corrected; see Fig. 1, left panel; note that the differences in RTs between old displays with high reward vs. old displays with low reward and the difference between new displays with high reward vs. old displays with low reward only approached significance: both *p*s > .20; see below).

**Fig. 2:**
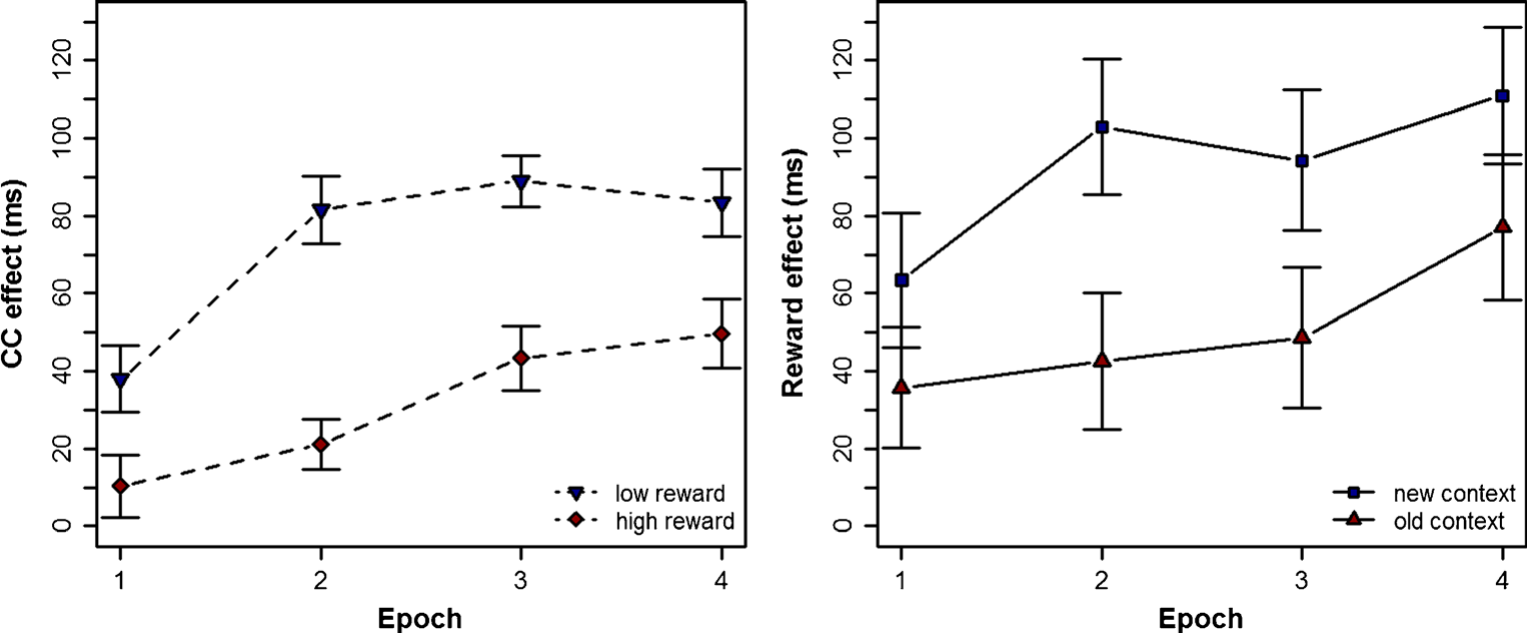
Interaction of context and reward. (Left) Context effects, calculated by subtracting individual mean reaction times (RTs) to old displays from RTs to new displays, shown separately for high- and low-reward trials. (Right) Reward effects, calculated by subtracting individual mean RTs to high-reward displays from those to low-reward displays, shown separately for old and new target–distractor contexts

Second, a comparison of contextual cueing—that is, RT for new minus RT for old displays between high- and low-reward trials (see Fig. 1, right panel)—revealed that for high-reward trials, the cueing effect was reliable from the third epoch onward [Epoch 1: 10 ms, *t*(24) = 0.6, *p* = .264; Epoch 2: 21 ms, *t*(24) = 1.6, *p* = .119; Epoch 3: 43 ms, *t*(24) = 2.6, *p* = .022; Epoch 4: 50 ms, *t*(24) = 2.8, *p* = .020]. For low-reward trials, by contrast, the effect had already gained significance in the first epoch (38, 82, 89, and 83 ms in Epochs 1–4, all *p*s < .020). Third, perhaps the most apt analysis that is diagnostic with regard to the Context × Reward interaction was to compare the contextual-cueing effects between high- and low-reward trials (left panel of Fig. 2), as well as the reward effect (RT high- minus RT low-reward trials) between old and new displays (right panel of Fig. 2). Figure 2 shows that although contextual cueing was overall larger in low- than in high-reward trials, reward effects were more pronounced overall for new than for old displays. In essence, reward effects for new displays were significant throughout the entire experiment (63, 103, 94, and 111 ms in Epochs 1–4, *p*s < .040), whereas the reward manipulation did not show a reliable effect for old displays (36, 43, 49, and 77 ms in Epochs 1–4, *p*s > .100).

## Discussion

In accordance with previous studies, the present experiment replicated contextual cueing, as indicated by faster RTs to old than to new displays. However, the most interesting results of the present study were that reward effects (1) were also measureable in new displays and (2) were actually smaller, rather than larger, in old displays. These results are difficult to explain by accounts assuming that reward enhances the learning of target–distractor contingencies—that is, *contextual cueing* (Tseng & Lleras, 2013). Instead, the results support the view that reward facilitates target location learning—that is, *probability cueing* (Jiang et al., 2013). This is not to say that reward plays no role in contextual cueing. Rather, there are two possibilities for explaining the significant Context × Reward interaction.

First, guidance by learned target–distractor arrangements and individual target positions may be additive, and reach a plateau after which RTs cannot be improved any further. Assuming that reward and contextual cueing occur at the very same time (a further analysis of RTs in the first epoch showed this to be the case; both effects emerged after just two cycles of presentation—that is, in Block 2 of the experiment), this would mean that once rewarded positions have been learned, contextual cueing cannot further improve visual search performance (or vice versa). The ultimate consequence is that context effects are smaller for high-reward trials.

Second, the effects of reward and contextual cueing could also be interpreted in the context of formal decision models, such as the Ratcliff diffusion model (RDM; Ratcliff, 1978). The model assumes that for binary decisions (e.g., left- vs. right-oriented targets), evidence is accumulated over time until a decision boundary is reached, which is followed by response execution. The drift rate of such an accumulation process may be influenced by some experimental manipulation—in the present experiment, reward or repeated target–distractor context—and an increase of this parameter yields shorter RTs. Zehetleitner and Müller (2010) have shown that differences in RTs due to a modulation of the drift rate are dependent on the overall duration of the decision process (modulations of the drift rate and associated performance gains are larger, the longer the overall decision process is). Applied to the present results, it is possible that contextual cueing has a smaller effect on high-reward trials because overall RTs are shorter on such trials, as compared to low-reward trials.

However, and in conclusion, the finding that contextual cueing is smaller in high-reward trials may also be taken as evidence for a less direct relationship between contextual cueing and reward. That is, given the available evidence (Tseng & Lleras, 2013) and after controlling for target repetition effects (present investigation), it is far more plausible to say that there is *no* relationship between configural learning and reward. Instead, it is more likely that monetary reward influences the learning of individual target locations (Jiang et al., 2013).
